# Application of CLIR-Based Post-Analytical Tools to Dutch NBS Data Demonstrates Its Potential Impact on the Performance of CPT1, GA-1, IVA and MSUD Screening in a Disorder-Specific Way

**DOI:** 10.3390/ijns12030070

**Published:** 2026-08-24

**Authors:** Nils W. F. Meijer, Rose E. Maase, Patricia L. Hall, Wouter F. Visser, Klaas Koop, Annet M. Bosch, M. Rebecca Heiner-Fokkema, Monique G. M. de Sain-van der Velden

**Affiliations:** 1 Department of Genetics, Section Metabolic Diagnostics, University Medical Center Utrecht, Lundlaan 6, 3584 EA Utrecht, The Netherlands; 2 Reference Laboratory for Neonatal Screening, Center for Health Protection, National Institute for Public Health and the Environment (RIVM), 3721 MA Bilthoven, The Netherlands; 3 Department of Laboratory Medicine and Pathology, Mayo Clinic, Rochester, MN 55905, USA; 4 Division of Metabolic Diseases, Department of Pediatrics, Emma Children’s Hospital, Amsterdam Gastroenterology Endocrinology and Metabolism, Amsterdam UMC, University of Amsterdam, Meibergdreef 9, 1105 AZ Amsterdam, The Netherlands; 5 Laboratory of Metabolic Diseases, Department of Laboratory Medicine, University of Groningen, University Medical Center Groningen, 9700 RB Groningen, The Netherlands

**Keywords:** collaborative laboratory integrated reports 1, postanalytical tools 2, covariate correction 3, CPT1 4, GA-I 5, IVA 6, MSUD 7, Dutch newborn screening 8

## Abstract

Newborn screening (NBS) for inborn errors of metabolism is challenged by high false-positive rates, which may lead to parental anxiety and increased healthcare costs associated with diagnostic follow-up. False-positive results often arise from changes in metabolite concentrations that mimic metabolic disorders as a consequence of differences in perinatal factors or nutritional status. To address this, Collaborative Laboratory Integrative Reports (CLIR) and the associated post-analytical tools (PATs) using multivariate interpretation and covariate-adjusted reference intervals may be used to improve specificity of NBS algorithms. In the current study, we examined whether CLIR can be applied to optimize the Dutch NBS program by reducing the false-positive rates. We developed and validated a CLIR-based PAT for CPT1 deficiency, GA-I, IVA and MSUD within the Dutch NBS program. Single-condition tools (SCTs) and multivariate approaches, including marker ratios and covariate adjustments, were evaluated for their ability to discriminate true- and false-positive referrals. For CPT1 deficiency, age-adjusted SCT combined with birthweight, location correction, and the C18:1/methionine ratio substantially reduced false positives. For GA-I, C3DC-based ratios improved specificity while preserving true-positive detection, potentially reflecting postnatal renal immaturity in some cases. For IVA, the dual scatter plot fully separated true- and false-positive referrals, highlighting the limitations of single-marker screening. For MSUD, differences between false-positive and true-positive cases were more pronounced, yet a similar number of false positives were still referred; valine-related markers contributed to false positives, while leucine and the Xle/Phe ratio better identified true positives. CLIR-based post-analytical tools enhanced NBS specificity through covariate-aware, multivariate interpretation. This provides important input for decision makers in both the Dutch NBS, as well as the NBS community worldwide.

## 1. Introduction

The Dutch newborn screening (NBS) program, in line with other NBS programs, has to date relied on a limited set of biochemical markers to detect a range of inherited metabolic disorders (IMDs) (the complete list of conditions included in the Dutch NBS is provided in Table S1). While highly effective for many conditions, this approach has inherent limitations, as single markers may lack sufficient sensitivity or specificity. Incorporating multiple markers and calculating ratios between markers can provide a more nuanced biochemical signature, improving screening performance and enabling the detection of subtle or atypical presentations [1,2,3]. In addition, the concentration of a given biomarker can be influenced by perinatal factors such as gestational age, birthweight, postnatal age, and sex, as well as physiological factors including parental nutritional status and genetic background [4,5,6,7,8], meaning that a “one size fits all” approach, or simple, step-wise age- and birthweight-adjusted cutoffs are often insufficient. When effects of perinatal and physiological factors can be generalized, tailoring marker panels and ratio thresholds to account for these variables enhances the predictive power of NBS and reduces the risk of unnecessary follow-up testing, without compromising NBS efficiency. By moving toward a more tailored strategy, screening programs can optimize early detection while minimizing the burden of false alarms on families and healthcare systems.

One strategy would be to use genetic screening to complement NBS by identifying specific pathogenic DNA variants associated with inherited disorders. However, currently, for most disorders there is insufficient evidence to establish a robust correlation between genetic variants and disease severity, rendering overall classification challenging. Therefore, integration of genetic screening into NBS programs is most clinically informative when biochemical data are available to guide decision making. A combined approach avoids false-negative and/or ambiguous NBS results which can result in asymptomatic individuals receiving a (genetic) diagnosis [9]. As an alternative strategy, static cutoffs may be replaced by multi-covariate-adjusted intervals that are generated based on regression analysis of large biochemical datasets thereby improving screening performance [10].

The Collaborative Laboratory Integrated Reports (CLIR) is an interactive web tool with clinical utilities, which bears relevance to these covariate effects on biomarker concentrations [10]. CLIR represents a practical strategy to optimize the interpretation of existing biochemical screening data. This provides an immediately applicable approach to improving screening performance within current NBS programs while genomic screening continues to evolve. Previously, we have shown that covariates significantly impact levels of markers currently used in the Dutch NBS program [7]. This justifies the necessity for covariate adjustment strategies. A key advantage of CLIR is that it moves beyond interpretation based on isolated marker concentrations. In routine NBS, referral decisions are often driven by one or a small number of primary markers, even though these metabolites are influenced by biological and pre-analytical factors such as age at sampling, birthweight, and broader metabolic variation. As a result, newborns without disease but with atypical biochemical profiles may still meet referral thresholds. By integrating multiple markers and adjusting for relevant covariates, CLIR enables interpretation of disease-associated abnormalities within their metabolic context, potentially reducing non-specific referrals.

Here, we report a retrospective study aimed at developing CLIR-based post-analytical tools (PATs) for a subset of inherited metabolic disorders (IMDs) included in the Dutch NBS program. The objective was to reduce false-positive screening results while maintaining the detection of affected newborns, thereby improving the positive predictive value (PPV) of the screening program. We assess the possible added value of CLIR for routine screening and provide detailed tool settings to serve as a resource for other programs that aim to integrate CLIR into their daily practice.

## 2. Materials and Methods

### 2.1. Study Population

Between 2018 and 2024, approximately 1 million newborns were screened in the Dutch national newborn screening program, with around 164,000–179,000 births occurring annually in the Netherlands. The average participation rate for the period 2018–2024 is 99.1%. All DBS samples were analyzed in five regional screening laboratories (since 2026, this has been reduced to three), which used the same analytical kit and harmonized protocols as part of the national NBS program. Heel-prick blood samples were collected on standardized filter paper cards by trained healthcare professionals and transported via routine postal/courier services to the designated regional screening laboratory. Data from 1 January 2018 to 31 December 2024 were extracted from the registry used by the Dutch National Institute for Public Health and the Environment, Department for Vaccine Supply and Prevention Programs (RIVM-DVP). Datasets were pseudonymized prior to use and contained the following elements: biological sex, gestational age, age at heel prick and analytical data of all markers included in the NeoBase2^TM^ Kit from Revvity (Turku, Finland).

### 2.2. Data Selection and Inclusion

The current study comprises samples from deidentified healthy controls and referrals for thirteen inherited metabolic disorders (listed in Table 1) in the Dutch newborn screening program between 1 January 2018 and 31 December 2024. For the present study, only the first heel-prick samples collected were included. Repeat samples were not included in the analyses. The target window for NBS sample collection in the Netherlands is between 3 and 7 days. Newborns sampled prior to this window (<3 days) were excluded from the study, as this group may include severely ill children that could limit generalizability of the results. Screening in the Netherlands is offered up to 6 months of age (183 days), therefore the included age range was between 3 and 183 days. Additional inclusion criteria were a birthweight between 500 and 6000 g and a gestational age between 23 and 44 weeks. Permission to use the data for this study was obtained from the commission Data applications Praeventis of the RIVM-DVP. Data were included from neonates whose parents did not object (before 2023) or consented (since 2023) to the use of heel-prick data for research purposes.

### 2.3. CLIR-PAT Development

**Figure 1:**
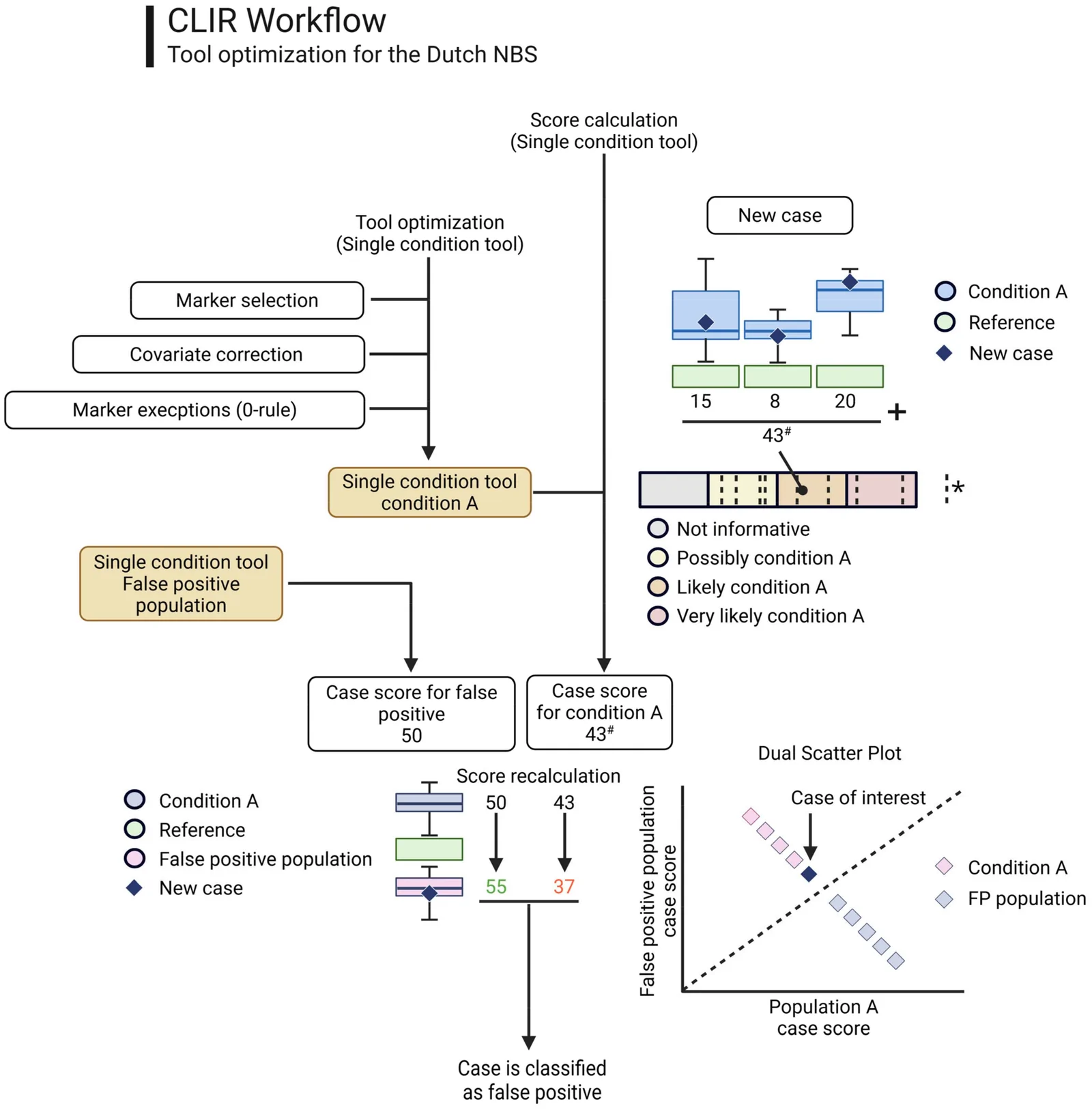
Flow scheme depicting the CLIR workflow. Following tool optimization including marker selection, covariate selection and the determination of potential marker exceptions (0-score rule), a marker score is calculated for each new case of interest using the optimized SCT. The marker score reflects the extent to which the concentration of the marker in the new case deviates from the reference population in a manner consistent with the deviation observed in the condition-specific population. The scores for all markers are then summed to generate an overall disease likelihood score. The disease likelihood score is compared with the scores obtained for all condition-specific cases in CLIR to define whether the case of interest is classified as not belonging, possibly belonging, likely to belong or very likely to belong to the condition-specific population. In this way, likelihood scores can be obtained not only for the condition of interest but also for a false-positive population or for other conditions that may result in a similar metabolic phenotype. If a case is classified as informative (falling within the possibly, likely or very likely categories) for both the condition of interest and the false-positive population based on the respective single-condition tools, the two scores can be compared using a dual scatter plot. In the dual scatter plot, scores are adjusted when the value of the case of interest for a marker (included in either of the single condition tools) falls within an interval that is unique to one of the two conditions being compared. In the example shown, the case of interest falls within an interval that is unique for the false-positive population. Consequently, the initial score for the false-positive population increases from 50 to 55, whereas the score for condition A decreases. The resulting scores for both conditions are then plotted in the dual scatter plot. Cases that fall above the dashed line, and therefore receive a higher score for the false-positive population than for condition A, are classified as false-positive cases. Cases falling below the dashed line are classified as condition A. Cases that fall on or close to the dashed line are classified as indeterminate. * Dashed lines represent scores obtained by true-positive cases of condition A within the CLIR database.

A general workflow was applied to optimize the PPV using the PAT in CLIR. Initially, single-condition tools (SCTs) were developed (Figure 1), utilizing the settings displayed in Supplementary Tables S2–S6. Single-condition tools generate a composite score obtained by summing the contributions of individual marker scores, thereby estimating the likelihood that an unseen case belongs to the condition of interest. Individual marker scores are derived from the covariate-adjusted value of the case under investigation and are determined by evaluating where this value lies relative to the covariate-corrected distributions observed in the reference and disease populations (i.e., if the case value deviates in a similar manner to the disease-specific population the maker score is higher). The contribution of each marker is further weighted according to its discriminatory capacity, reflecting how informative that marker is in distinguishing between the reference and disease groups. Markers exhibiting greater separation between the two populations contribute more strongly to the overall score as compared to markers with substantial overlap. The summed score obtained is compared with the scores of all active cases in the database that contain values for the markers and covariates included in the single-condition tool (markers and covariates included in each tool are displayed in Supplementary Tables S2–S6). A case is classified as informative for the condition when its score exceeds a predefined threshold, which can be determined either by an absolute point-based cutoff or by percentile ranking. When percentile ranking is applied, newly analyzed cases are assigned a percentile rank rather than an absolute score, allowing comparison with the distribution of scores observed in the active case database. When the latter option is selected new cases receive a percentile rank rather than a point-based score for comparison The selection of informative markers was based on the discriminatory power (overlap ≤ 20%) between the condition-specific and reference intervals in combination with the availability of sufficient informative cases (concentration for a marker must be available for at least 80% of the uploaded cases in CLIR). Tools built on too few informative cases may generalize poorly and potentially lose effectiveness on unseen data. The only two exceptions in the current manuscript are the addition of the leucine/alanine ratio (available for 65% of MSUD cases) to the MSUD SCT and the inclusion of markers in the FP C5 SCT for IVA (44% of cases). Regarding MSUD, the leucine/alanine ratio was one of the most important marker ratios for discriminating MSUD due to the in vivo relationship between branched-chain amino acid catabolism and alanine production (Figure S1) [11]. With regard to the FP C5 SCT, C5 contained a substantially higher number of cases (*n* = 112) compared to many of the other informative markers (*n* = 76). As a result, exclusion (based on ≥80% of total available cases) would have led to the inclusion of very few markers potentially limiting the validity of the tool. Markers with extremely low baseline values, which were not associated with the condition of interest, were excluded from the tool as small absolute differences (from 0.01 to 0.02) can result in large differences in marker ratios that are unlikely to be biologically relevant.

Covariate selection was based on identifying the covariates that produced the greatest discrimination between reference and condition-specific covariate intervals after correction within CLIR. Since certain covariate combinations were only available for a small subset of the markers included in the tool, the combination of BW, age, and location was found to be the most discriminatory in most cases. In addition, covariate adjustments must be applied consistently across all markers included in a tool to avoid false results originating from ratios between markers that are corrected utilizing different covariates.

Next, the tuner functionality was used to identify markers for which part of the covariate-adjusted reference range was discriminatory (non-overlapping) for either the false- or true-positive populations. When this occurred, the marker was added as a negative regulator (0-score, marker exception). As a general rule, marker exceptions should not result in a non-informative score for true-positive cases for the conditions of interest. If the SCT functionality did not yield sufficient improvement for the disease of interest, SCTs for both the false-positive and true-positive population were combined into a dual scatter plot. In this approach, the initial scoring from both tools is adjusted when one or more covariate-corrected marker levels falls within a region exclusive to either the true or false population. Each case thus receives a score reflecting its likelihood of belonging to either the true or false population, based on all informative cases included in both SCTs. To prevent overfitting, the tools were generated based on the available data in CLIR and subsequently validated using Dutch NBS data on true- and false-positive cases. In the cumulative dataset in CLIR, one Dutch case of CPT1 and two Dutch cases of GA-I are included (the total number of cases used in each tool can be found in Supplementary Tables S2–S6).

### 2.4. Rationale for the Selection of Disorders Presented in the Results Section

In the current study, we examined whether CLIR and its associated PAT could be used to improve the PPV for all IMD’s currently included in the Dutch NBS program (Table 1). Data for PA and MMA are not presented here, as they have been reported previously (MMA manuscript submitted) [12]. With regard to TT1, the majority of the cases included in CLIR were collected before the Neobase 2^TM^ kit was commonly integrated into NBS programs. Succinylacetone concentrations produced with the Neobase 2^TM^ kit are significantly different from those produced with the Neobase 1TM kit; therefore, two method-specific variants of the marker (SUAC) are defined in CLIR. Due to mutual exclusivity, inclusion of SUAC measured with the Neobase 2^TM^ kit (the only marker used in the Dutch first-tier algorithm for TT1) in the PAT led to the exclusion of the majority of informative positive cases for TT1 from the tool (65 out of 75). This resulted in an SCT based on only 10 positive cases which represents the total population in CLIR insufficiently. In addition, succinylacetone is not a pathognomonic marker for TT1, as it can also be elevated in individuals with MAAI deficiency [13]. Furthermore, it is unclear whether uploaded cases of TT1 in CLIR have been genetically confirmed. TT1 was therefore not considered in this study. Data for PKU were also excluded as no further improvement was needed for our program as only 1 false-positive case was found during the study period. Data for VLCAD (too few false positives), LCHAD (too few false positives), MCAD (too few false positives) and 3-MCC (heterogenous nature), HMG-CoA lyase (no referrals) deficiency, and MCD (no referrals) deficiencies are omitted.

## 3. Results

PATs for the diagnosis of CPT1 deficiency, GA-I, IVA and MSUD were developed and validated. A detailed overview of the settings for each tool is included in Supplementary Tables S2–S6.

### 3.1. CPT1 Deficiency

**Figure 2:**
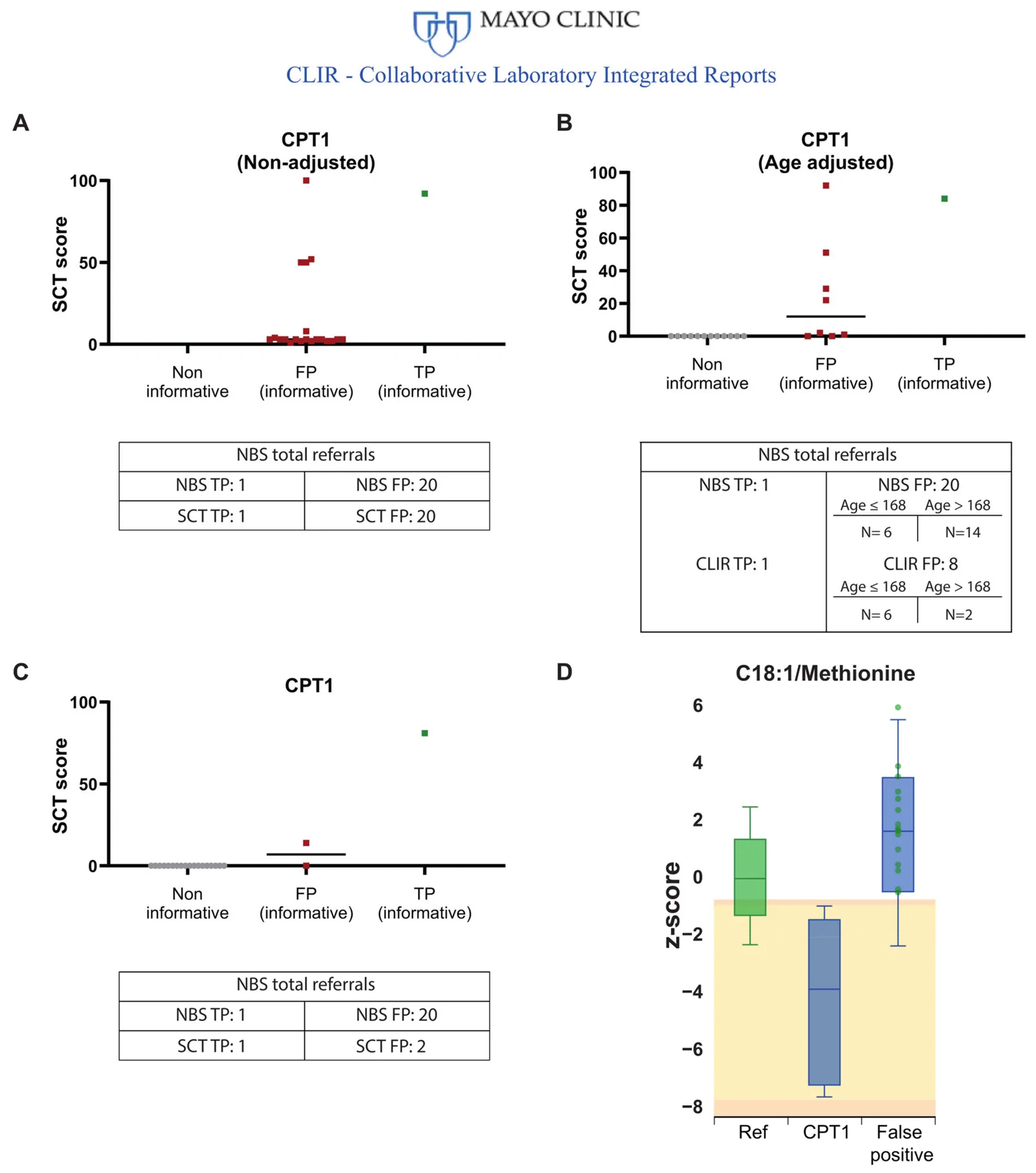
(**A**–**C**) Scatterplot indicating the normalized (between 0 and 100) score attributed by the non-adjusted SCT (**A**), the age-adjusted SCT (**B**), and the optimized (including age, birthweight and location adjustments and incorporation of additional markers) SCT for CPT1 deficiency (**C**) to each referral for CPT1 deficiency (*n* = 21), with green indicating true-positive cases of CPT1 deficiency, red indicating false positives that were categorized as being informative and gray indicating false-positive cases that were categorized as being non-informative by the SCT. The table indicates the number of TP and FP cases based on the current NBS protocol (top) and CLIR SCT (bottom). Settings for the SCTs can be found in Supplementary Table S2. (**D**) Boxplot comparing the covariate-corrected reference intervals (based on age, birthweight and location correction) of the C18:1/methionine ratio for the reference, CPT1 deficiency-positive and false-positive population in CLIR.

In the Dutch NBS program, the risk of CPT1 deficiency is assessed by measuring free carnitine (C0) and determining the C0/(C16 + C18) ratio [14], with referral only if both markers are elevated. In recent years, the number of false positives for CPT1 deficiency has increased substantially. The majority of the false-positive cases originate from newborns who receive the heel prick at an older age. C0 levels increase with age, while C16 and C18 levels decrease with age (Supplementary Figure S2) [15]. To distinguish effects attributable to age from those arising from differences between the Dutch NBS and CLIR algorithms, we first configured the SCT to replicate the Dutch NBS algorithm. As shown in Figure 2A, this produced highly similar results, with 20 out of the 20 false-positive cases being classified as informative for CPT1 deficiency (settings: Supplementary Table S2). Next, we assessed the impact of age adjustment by developing an SCT based solely on the age-adjusted C0 and C0/(C16 + C18) intervals (Figure 2B and Supplementary Table S2). This approach reduced the number of false positives classified as informative for CPT1 deficiency from 20 to 8. As expected, this reduction was primarily observed in cases for which the heel prick was obtained outside of the target window. We additionally evaluated the effects of age adjustment on true-positive CPT1 deficiency cases. As illustrated in Supplementary Figure S3, age adjustment did not increase the overlap between the C0 and C0/(C16 + C18) intervals of CPT1 deficiency and the reference population (Supplementary Figure S3).

After confirming the effectiveness of age adjustment, we subsequently explored the broader SCT capabilities. This included applying additional correction factors—birthweight (BW) and location adjustments—and incorporation of additional markers. These enhancements led to a substantial increase in the PPV when compared to the current NBS algorithm (Table S2 and Figure 2C). Optimization improved the PPV from 5% to 33%, representing a 7-fold increase and a reduction in false-positive referrals from 20 to 2. The reduction in false-positive referrals was primarily driven by the inclusion of the C18:1/methionine ratio as a negative regulator (0—marker score rule), that was established using the tuner utility based on the non-overlapping region between the false-positive (FP C0 H (high)) and CPT1 deficiency-positive populations in CLIR (Figure 2D). Overall, our data demonstrate that utilization of CLIR, along with covariate-corrected reference intervals, has significant potential to improve the accuracy of CPT1 deficiency screening compared with the current Dutch CPT1 deficiency NBS algorithm.

### 3.2. GA-I

**Figure 3:**
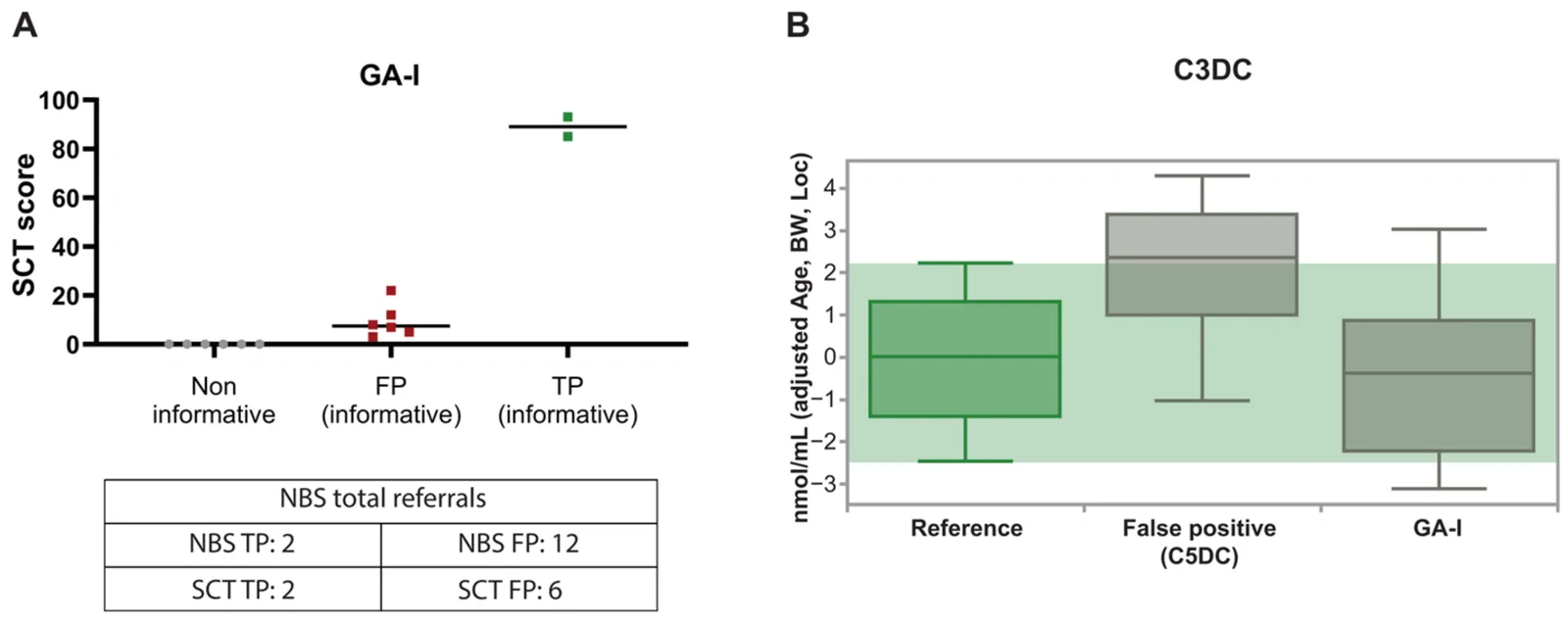
(**A**) Scatterplot indicating the normalized (between 0 and 100) score attributed by the optimized SCT for GA-1 to each referral for GA-I (*n* = 14), with green indicating true-positive cases of GA-I, red indicating false positives that were categorized as being informative and gray indicating false-positive cases that were categorized as being non-informative by the SCT. Settings for the SCT can be found in Supplementary Table S3. (**B**) Boxplot comparing the covariate-corrected reference intervals (based on age, birthweight and location correction) of C3DC for the reference, FP C5DC, and GA-I TP populations in CLIR.

In the Dutch NBS, GA-I is primarily identified by measuring of glutarylcarnitine (C5DC) [14]. The current C5DC cutoff used in the Dutch screening program is 0.35 µmol/L. Identification of GA-I is challenging because some patients exhibit a mild biochemical phenotype, so-called ‘low excretors’, lacking a significant elevation in C5DC [8]. Moreover, since the NeoBase 2TM kit C5DC assay employs a non-derivatized flow injection methodology, C5DC measurements may be susceptible to interference from non-specific hydroxyl-containing species (C6OH). Consequently, distinguishing these patients from healthy individuals with naturally higher baseline C5DC levels or from patients with other factors such as renal insufficiency is difficult [16]. This has resulted in a poor predictive value for GA-I in the Dutch NBS (PPV 14%: 2 TP and 12 FP). Simply increasing the C5DC cutoff is not feasible, as it would risk missing patients with the low excretor phenotype. We therefore utilized CLIR to investigate whether incorporating additional markers could improve GA-I screening while maintaining sensitivity to low excretor cases; we found that the number of false positives could be significantly reduced from 12 to 6, with the PPV improving from 14% to 25%. (Figure 3A, SCT settings are displayed in Supplementary Table S3.) This improvement was largely driven by including the C3DC/ornithine and C3DC/alanine ratios as a negative regulator (0—marker score rule) (Supplementary Figure S4A). As shown in Figure 3B (Supplementary Figure S4B for the Dutch NBS data on GA-I), the discriminatory capacity of C3DC-based ratios is largely dominated by C3DC itself, indicating that although these ratios contributed to the optimized algorithm, their added value beyond C3DC alone appears to be limited. Interestingly, C3DC primarily discriminates the false-positive population rather than the GA-I-specific population from the reference population.

### 3.3. IVA

**Figure 4:**
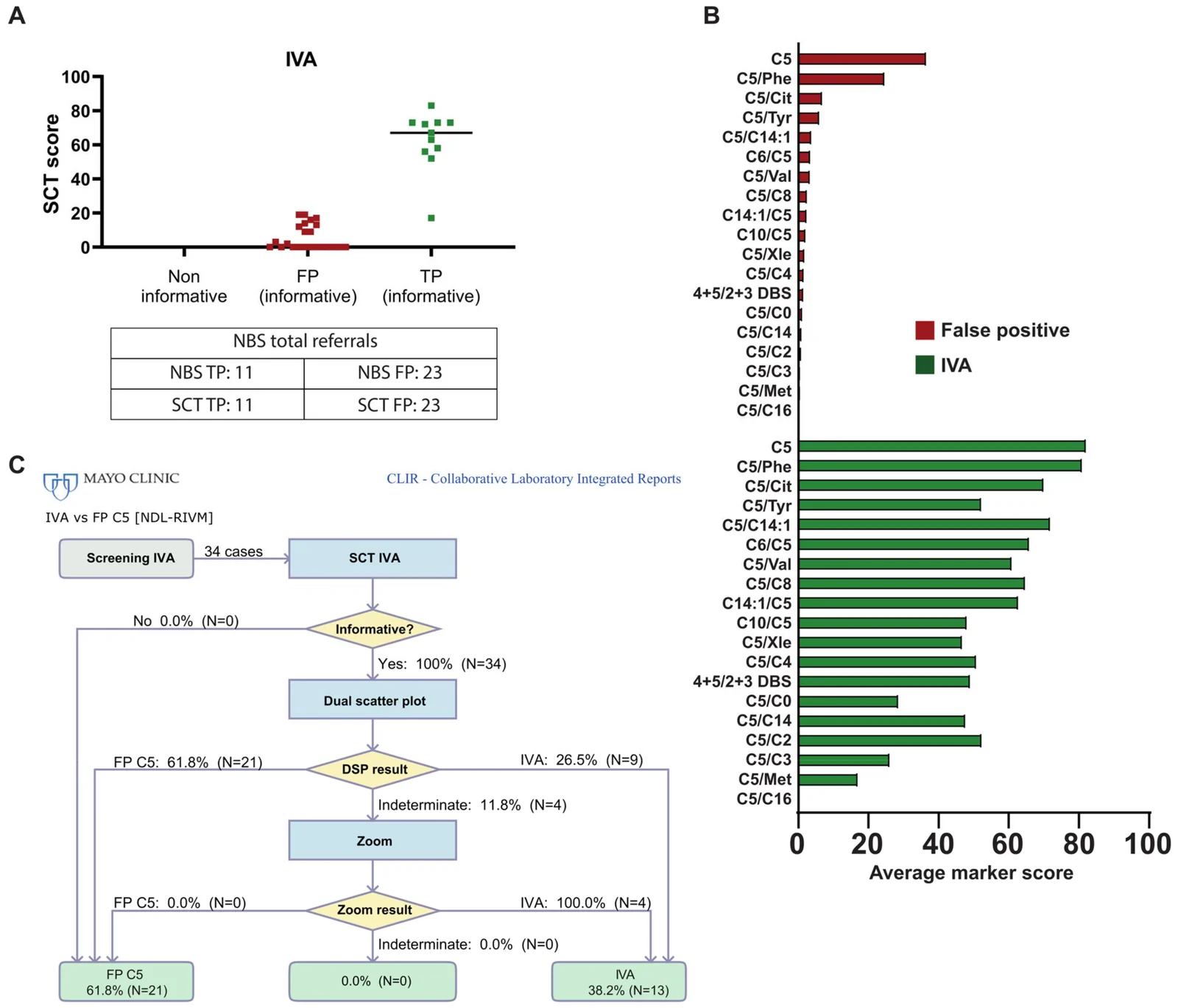
(**A**) Scatterplot indicating the normalized (between 0 and 100) score attributed by the optimized SCT for IVA to each referral for IVA (*n* = 34), with green indicating true-positive cases of IVA, red indicating false positives that were categorized as being informative and gray indicating false-positive cases that were categorized as being non-informative by the SCT. The table indicates the number of TP and FP cases based on the NBS and CLIR SCT. Settings for the SCT can be found in Supplementary Table S4. (**B**) Bar graph indicating the average scores obtained for each marker included in the SCT for the false-positive (red bars) and IVA (green bars) populations. (**C**) Flow scheme depicting the output of the dual scatter plot for referrals for IVA derived from the Dutch NBS program. The CLIR dual scatter plot compares two conditions by assigning each case a score for both conditions based on the single-condition tool (SCT) score. Unlike the SCT, which classifies cases relative to the reference and true-positive (TP) populations, the dual-condition approach evaluates whether cases fall within condition-specific areas of the interval unique to either of the two populations that are being compared (in this case the IVA and C5 FP populations). Scores are recalculated using all markers from both tools and the score is adapted only when a marker value falls within an interval that is unique to either the true-positive (TP) or false-positive (FP) populations for one of the conditions. Cases that cannot be unambiguously assigned are classified as indeterminate. The resulting updated SCT scores are displayed in the dual scatter plot and used to classify each case as TP, FP, or indeterminate. The zoom functionality generates a secondary dual scatter plot containing only the indeterminate cases, enabling further refinement and classification of these ambiguous cases.

In the Dutch NBS, IVA is primarily identified by measuring isovalerylcarnitine (C5) [14]. While the SCT for IVA did not reduce the number of false-positive cases; scoring between false-positive referrals and true-positive IVA cases differed significantly with the exception of one true-positive case (Figure 4A, settings: Supplementary Table S4). This prompted us to identify which markers were driving the difference in scores between the IVA population and false-positive cases that still assigned informative scores for IVA. To address this, we compared the average scores for each marker included in the tool across both populations. Interestingly, C5 (used as a primary marker), showed the smallest difference between the true-positive and false-positive groups (Figure 4B). This observation was to be expected, as screening based on a single variable inherently preselects healthy individuals who naturally exhibit elevated levels of this marker, highlighting the limitations of a one-dimensional screening approach.

We then applied the dual scatter plot to further discriminate between both populations. In this approach, scores from the SCTs for FP C5 and IVA are adjusted based on the non-overlapping regions between the conditions for each included marker. This adjustment reduced the number of false positives from 23 to 2, with the PPV improving from 32% to 85% (Figure 4C, settings FP5 SCT: Supplementary Table S5). The markers driving these score adjustments may differ across datasets and evaluation settings.

### 3.4. MSUD

**Figure 5:**
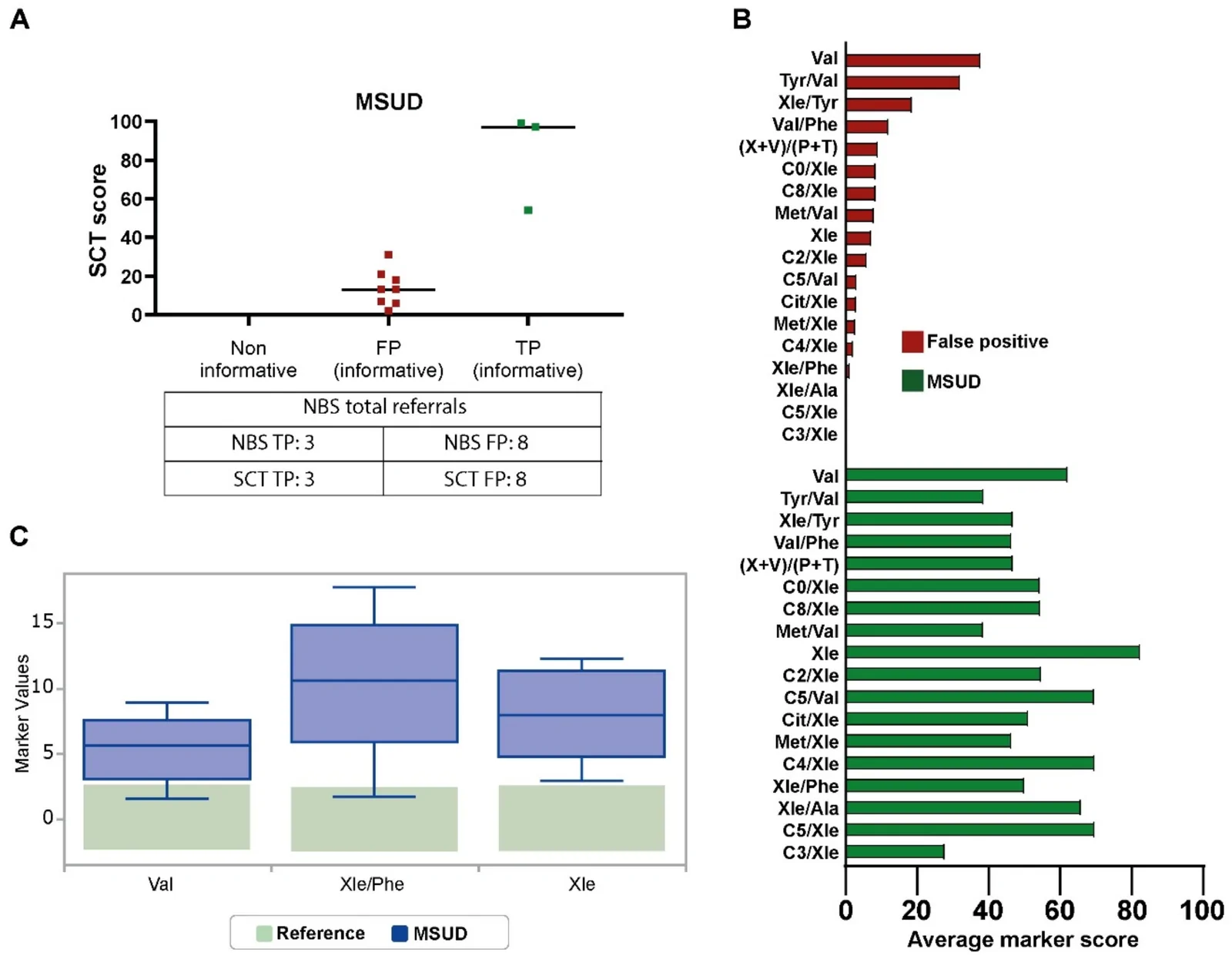
(**A**) Scatterplot indicating the normalized (between 0 and 100) score attributed by the optimized SCT lacking valine and ratios with valine to each referral for MSUD (*n* = 11), with green indicating true-positive cases of MSUD, red indicating false positives that were categorized as being informative and gray indicating false-positive cases that were categorized as being non-informative by the SCT. The table indicates the number of TP and FP cases based on the NBS and CLIR SCT. Settings for the SCT can be found in Supplementary Table S6. (**B**) Bar graph indicating the average scores obtained for each marker included in the SCT for the false-positive (red bars) and MSUD (green bars) populations. (**C**) Boxplot comparing the covariate-corrected reference intervals (based on age, birthweight and location correction) of valine, leucine and the leucine/phenylalanine ratio for the reference and MSUD-positive populations in CLIR.

Based on a previous evaluation of the NBS for MSUD in the Netherlands, the Xle/Phe ratio was added resulting in a screening algorithm based on Xle, valine and the XLE/PHE ratio [14,17]. Here, we examined whether we could further improve the PPV for MSUD using CLIR. To this end, we developed an SCT incorporating all informative markers associated with MSUD (settings are displayed in Supplementary Table S6). Although all true-positive cases received higher scores than the false-positive cases, all cases were still classified as informative for MSUD (Figure 5A). To identify which markers contributed to the informative scores in both groups, we evaluated the contribution of each individual marker to the CLIR score calculation (Figure 5B) This analysis showed that valine and valine-related ratios accounted for a substantial proportion of the total score in the false-positive population, whereas their contribution was much smaller in MSUD cases. This likely reflects the fact that covariate-corrected intervals for valine lie in close proximity to the reference population, while leucine and the Xle/Phe ratio show greater separation (Figure 5C). Together, these findings suggest that valine may be less suitable for discriminating between individuals who naturally exhibit higher valine levels in the reference population.

## 4. Discussion

In this study, we developed and validated CLIR-based PATs for four disorders included in the Dutch NBS program (CPT1 deficiency, GA-I, IVA, and MSUD). Overall, these tools improved the distinction between true-positive and false-positive referrals. The extent and mechanism of improvement differed by disorder, but across all conditions, the results support the value of combining covariate-adjusted reference intervals with multivariate metabolite interpretation to increase screening specificity.

For CPT1 deficiency, age at sampling was the major driver of false-positive referrals [7,15]. Configuring the SCT to replicate the Dutch NBS algorithm showed that age adjustment alone substantially reduced false positives, particularly in newborns sampled outside the target heel-prick window, without increasing overlap with true-positive cases. Further optimization—including birthweight and location correction and the addition of the C18:1/methionine ratio —improved the PPV by distinguishing false-positive children with a high C0-carnitine from true CPT1 deficiency cases. The discriminatory power of the C18:1/methionine ratio was primarily due to severe reductions in C18:1, consistent with early studies showing that impaired CPT1 activity limits the conversion of oleoyl-CoA to oleoylcarnitine despite sufficient carnitine [18]. Based on our previous study, adjustment for gestational age may also be considered. However, as an insufficient number of CPT1 deficiency cases contained gestational age data, this covariate was not included in our tool [1]. These findings demonstrate that CLIR-based PATs enhance screening both by identifying disease-specific patterns and by recognizing biochemical profiles inconsistent with disease.

Due to the relatively high number of false positives, confirmatory diagnostics for suspected GA-I are frequently employed. Simply raising the C5DC cutoff for GA-1 would reduce sensitivity for clinically relevant cases [8]. Our results show that CLIR improves specificity while maintaining true-positive detection, primarily through C3DC-based ratios as negative regulators. The discriminatory effect was largely driven by C3DC itself, separating false positives rather than GA-I cases, and may also highlight trends distinguishing the false-positive population from both reference and disease-positive groups. Notably, elevated C3DC and C5DC in false positives may reflect postnatal renal immaturity/insufficiency, consistent with previous observations that acylcarnitines correlate negatively with estimated glomerular filtration rate (eGFR) [19]. This suggests a common, biologically plausible cause for false-positive elevations.

For IVA full separation was only achieved using the dual scatter plot. The primary marker C5 showed a minimal difference between true- and false-positive groups, reflecting that single-marker-based referral inherently enriches for individuals with naturally or environmentally elevated levels. This illustrates the limitation of one-dimensional screening for conditions such as IVA (and GA-I), which rely on a single metabolite rather than ratios. By evaluating multiple markers and their ratios, we place condition-associated metabolites within the broader metabolic context (e.g., using the NeoBase^TM^ 2 kit), helping to account for variations due to supplementation, liver or kidney function, or other non-disease factors, as exemplified by the C5/C2 ratio [3,19,20,21].

For MSUD, differences between false-positive and true-positive cases were more pronounced using the SCT, yet the PPV remained unchanged. We showed that adding markers does not automatically enhance performance. The SCT for true-positive cases scored higher than false positives, but all referrals remained informative. Analysis of marker contributions indicated that valine and valine-related ratios largely drove scores in false positives, whereas leucine and the Xle/Phe ratio better separated true-positive cases. This suggests that valine may add noise rather than diagnostic value, likely because its covariate-corrected intervals overlap with the reference population. These results highlight that marker selection in PATs should prioritize discriminatory power over metabolic relevance alone. Further optimization could potentially be obtained by comparing the MSUD-positive population to the group of children that receive total parenteral nutrition (TPN). However as significant difference exists in compositions of TPN between countries, and data for the Dutch NBS ‘TPN population’ are not available, this has not been explored.

Taken together, the present results show that CLIR can improve screening performance, but that the mechanisms through which these improvements can be achieved are disorder-specific. This suggests that implementation of CLIR-based PATs in NBS should not follow a uniform model but should instead be tailored to the characteristics of each condition and country.

Several limitations should be noted. Retrospective analysis limits assessment of real-world impact on referral rates and follow-up. A further limitation is that referral definitions and diagnostic confirmation vary internationally, which can affect CLIR classification, particularly for special populations (e.g., TPN recipients) or cases with single severe mutations. Additionally, our findings should be interpreted within the context of the Dutch newborn screening program, meaning that validation in other national screening programs will be necessary before broader implementation can be recommended. Despite these limitations, the clinical relevance of these findings is clear. Any strategy that reduces false-positive referrals without compromising case detection therefore has immediate practical value.

In conclusion, CLIR-based post-analytical tools improved the interpretation of referrals for CPT1 deficiency, GA-I and IVA, while the benefit was limited for others, such as MSUD within the Dutch NBS program. The main benefit was improved specificity through disorder-specific, covariate-aware, multivariate interpretation of metabolic profiles. These findings support further prospective validation and disorder-specific implementation of CLIR as part of routine NBS practice.

## Figures and Tables

**Table 1:** Conditions included in the study (detected by acylcarnitine and amino acid analysis).

Condition	Abbreviation	OMIM-Code(s)
Carnitine palmitoyl transferase 1 deficiency	CPT1 deficiency	255120
Glutaric acidemia I	GA-I	231670
Isovaleric acidemia	IVA	243500
3-Methylcrotonyl-CoA carboxylase deficiency	3MCC	210200, 210210
3-Hydroxy-3-methylglutaryl-CoA lyase deficiency	HMG-CoA lyase deficiency	246450
Multiple carboxylase deficiency	MCD deficiency	253260
Medium-chain acyl-coenzyme A dehydrogenase deficiency	MCAD deficiency	607008
Long-chain 3-hydroxyacyl-CoA dehydrogenase deficiency	LCHAD deficiency	609016
Maple syrup urine disease	MSUD	248600
Propionic acidemia	PA	606054
Methylmalonic aciduria	MMA	251000, 251100, 251110, 277400, 277410, 608419
Tyrosinemia type 1	TT1	276700
Very long-chain acyl-CoA dehydrogenase deficiency	VLCAD	609575

## Data Availability

The datasets presented in this article are not readily available because the data are part of an ongoing study. Requests to access the datasets should be directed to the corresponding author.

## Supplementary Materials

The following supporting information can be downloaded at: https://www.mdpi.com/article/10.3390/ijns12030070/s1, Table S1: Conditions included in the Dutch newborn screening program; Table S2: configuration of the SCTs for CPT1 deficiency; Table S3. configuration of the SCT for GA-I; Table S4. configuration of the SCT for IVA; Table S5. configuration of the SCT for FP C5; Table S6. configuration of the SCT for MSUD; Figure S1. Boxplot comparing the covariate-corrected reference intervals (based on age, birthweight and location correction) of the alanine concentration and the leucine/alanine ratio for the reference and MSUD-positive populations in CLIR; Figure S2. Moving percentiles derived from CLIR, indicating the impact of age on free carnitine (C0), C16 and C18 levels (nmol/mL) in newborn infants. Retrieved from https://clir.mayo.edu/ (accessed on the 7 of April 2026); Figure S3. Age adjustment comparison chart showing the unadjusted and age-adjusted corrected intervals (upper) or age-adjusted corrected intervals only (lower) for C0 and the C0/(C16 + C18) ratio in the reference and CPT1 deficiency-positive population; Figure S4. (A) Boxplot comparing the covariate corrected reference intervals (based on age, birthweight and location correction) of the C3DC/Ornithine ratio and the C3DC/Alanine ratio for the reference, false-positive C5DC, and GA-I-positive populations in CLIR. (B) Scatterplot indicating the concentration of C3DC and C5DC for false-positive (FP) and true-positive (TP) cases of GA-I in the Dutch NBS program.

## Abbreviations

The following abbreviations are used in this manuscript:

NBS	Newborn screening
CLIR	Collaborative Laboratory Integrative Reports
PAT	Post-analytical tools
CPT1 deficiency	Carnitine palmitoyl transferase 1 deficiency
GA-I	Glutaric acidemia I
IVA	Isovaleric acidemia
3MCC	3-Methylcrotonyl-CoA carboxylase deficiency
HMG-CoA lyase deficiency	3-Hydroxy-3-methylglutaryl-CoA lyase deficiency
MCD deficiency	Multiple carboxylase deficiency
MCAD deficiency	Medium-chain acyl-coenzyme A dehydrogenase deficiency
LCHAD deficiency	Long-chain 3-hydroxyacyl-CoA dehydrogenase deficiency
MSUD	Maple syrup urine disease
PA	Propionic acidemia
MMA	Methylmalonic aciduria
TT1	Tyrosinemia type 1
VLCAD	Very long-chain acyl-CoA dehydrogenase deficiency
SCT	Single condition tool
IMDs	Inherited metabolic disorders
PPV	Positive predictive value
FP	False positive
TP	True positive
eGFR	Estimated glomerular filtration rate
TPN	Total parenteral nutrition
