# Supplementary material for: Application of CLIR-Based Post-Analytical Tools to Dutch NBS Data Demonstrates Its Potential Impact on the Performance of CPT1, GA-1, IVA and MSUD Screening in a Disorder-Specific Way
Source: Int J Neonatal Screen. 2026 Aug 24;12(3):70. doi: 10.3390/ijns12030070 (PMC13607394; doi:10.3390/ijns12030070)
Supplement: Supplementary file 1 [file IJNS-12-00070-s001.zip › IJNS-4401951-supplementary.pdf]

Supplementary information

Table S1. Conditions included in the Dutch newborn screening program

Condition	Configuration
Endocrine disorders	Congenital hypothyroidism (CH), Congenital adrenal hyperplasia (CAH/AGS)
Metabolic disorders	3-Methylcrotonyl-CoA carboxylase deficiency (3-MCC), Adrenoleukodystrophy (X-ALD), Biotinidase deficiency, Carnitine palmitoyltransferase I deficiency (CPT1), Classical galactosaemia (GALT), Galactokinase deficiency (GALK), Glutaric aciduria type I (GA-I), HMG-CoA lyase deficiency, Isovaleric acidaemia (IVA), Long-chain 3-hydroxyacyl-CoA dehydrogenase deficiency (LCHADD), Maple syrup urine disease (MSUD), Medium-chain acyl-CoA dehydrogenase deficiency (MCADD), Methylmalonic acidaemia (MMA), Multiple carboxylase deficiency (MCD), Mucopolysaccharidosis type I (MPS I), Phenylketonuria (PKU), Propionic acidaemia (PA), Tyrosinaemia type I (TYR-I), Very long-chain acyl-CoA dehydrogenase deficiency (VLCADD)
Blood disorders	Sickle cell disease, Alpha-thalassaemia (HbH disease), Beta-thalassaemia major
Respiratory disorder	Cystic fibrosis (CF)
Immune disorder	Severe combined immunodeficiency (SCID)
Neuromuscular disorder	Spinal muscular atrophy (SMA)

Table S2. configuration of the SCTs for CPT1 deficiency

Condition	Configuration	Included markers	Covariate corrections	Marker exceptions	Tool based on N cases
CPT1 (Non-adjusted)	Increasing Regular Min/Max Plot differentiators<br><br>Guidelines: Default points	**High Markers**<br>C0<br>C0/(C16+C18)	N.A.	N.A.	n = 33<br><br>(Including 1 Dutch case)

Condition	Configuration	Included markers	Covariate corrections	Marker exceptions	Tool based on N cases
CPT1 (Age-adjusted)	Increasing Regular Min/Max Plot differentiators<br><br>Guidelines: Default points	**High Markers**<br>C0<br>C0/(C16+C18)	Age	N.A.	n = 33<br><br>(Including 1 Dutch case)

Condition	Configuration	Included markers	Covariate corrections	Marker exceptions	Amount of cases included
CPT1	Increasing Weighted Min/Max Plot differentiators<br><br>Guidelines: Default Points	**High Markers**<br>C0<br>C0/(C16+C18)<br>C0/C16<br>C0/C18<br>C0/Phe<br>C0/Val<br>C0/Xle<br>C0/Met<br>C3/C16<br>C4/C18:1	Age<br>BW<br>Location	C18:1/Met	n = 31<br><br>(Including 1 Dutch case)
		**Low Markers**<br>(C16+C18)/C2<br>(C16+C18:1)/C2<br>C14/C0<br>C14:1/C0<br>C16/C2<br>C18:1/C0<br>C4/C0<br>C16/Tyr<br>C18<br>C18/C2<br>C2/C0<br>C18:1/Tyr<br>C5/C0<br>(C16+C18:1)/C3<br>C16/Cit<br>C18/C3<br>C18:1/C2<br>C16/Phe<br>Cit/C0<br>C16<br>C18:1/C3<br>C18:1/Phe<br>C18:1/Xle<br>C18:1<br>C18:1/Cit<br>C18:1/Met			

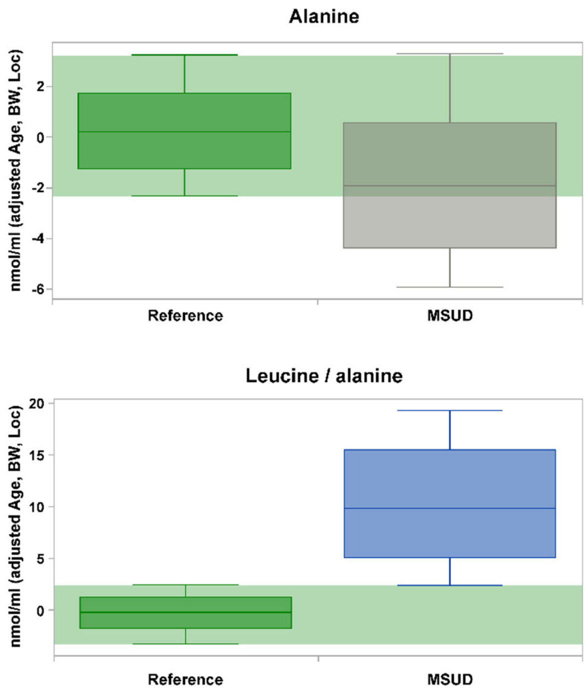

Figure S1. Boxplot comparing the covariate-corrected reference intervals (based on age, birthweight and location correction) of the alanine concentration and the leucine/alanine ratio for the reference and MSUD-positive populations in CLIR.

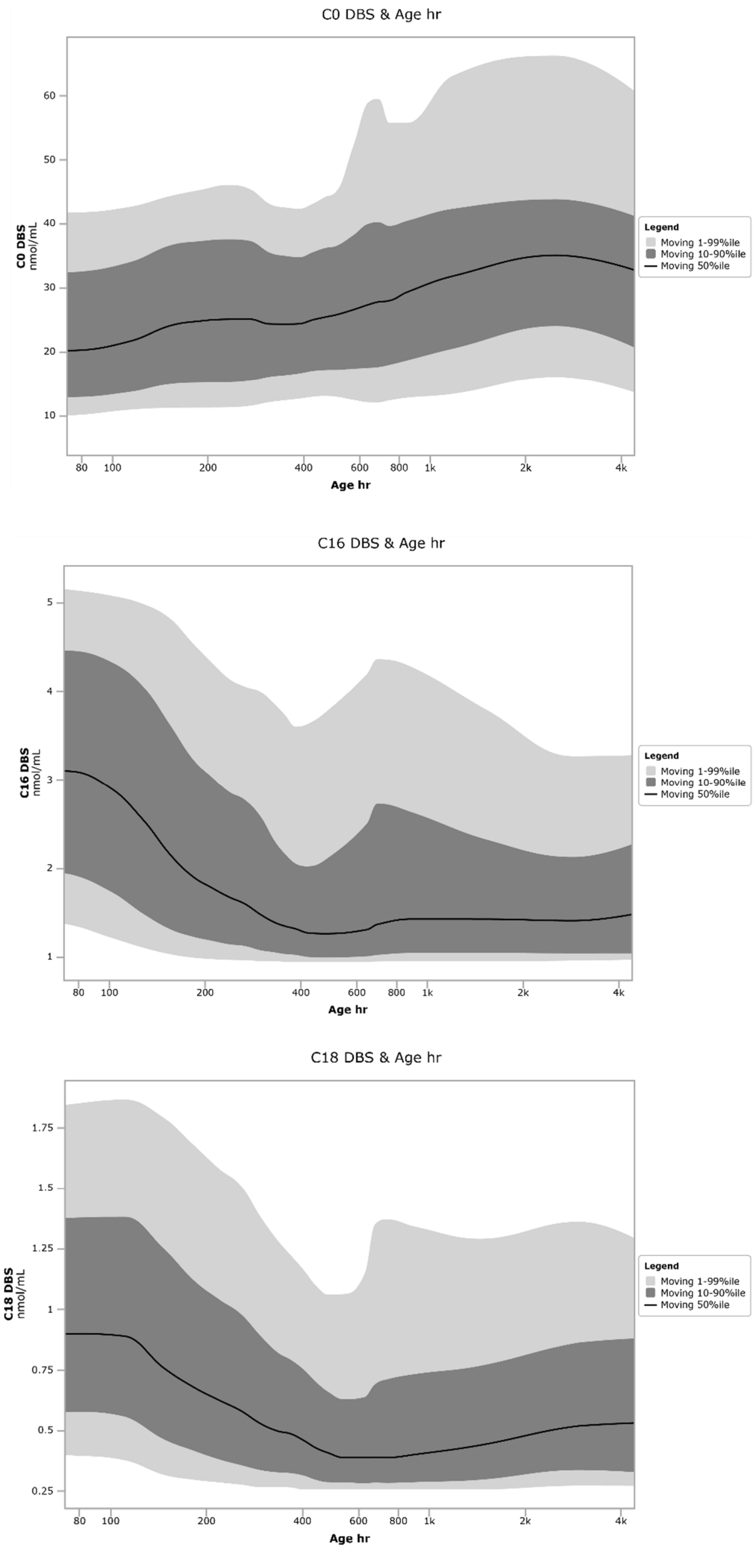

Figure S2. Moving percentiles derived from CLIR, indicating the impact of age on free carnitine (C0), C16 and C18 levels (nmol/mL) in newborn infants. Retrieved from https://clir.mayo.edu/ (accessed on the 7 of April 2026).

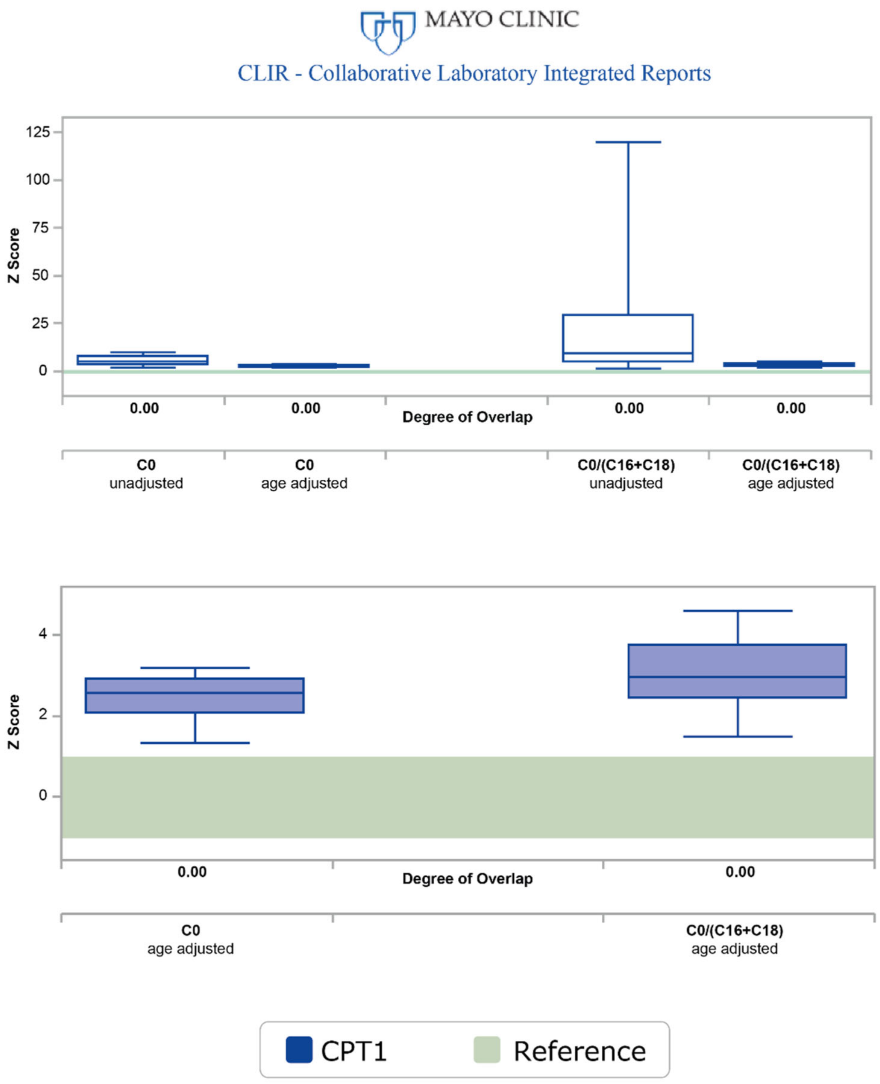

Figure S3. Age adjustment comparison chart showing the unadjusted and age-adjusted corrected intervals (**upper**) or age-adjusted corrected intervals only (**lower**) for C0 and the C0/(C16 + C18) ratio in the reference and CPT1 deficiency-positive population

Table S3. configuration of the SCT for GA-I.

Condition	Configuration	Included markers	Covariate corrections	Marker exceptions	Amount of cases included
GA-I	Increasing Weighted Min/Max Plot differentiators<br><br>Guidelines: Default Points	**High Markers**<br>C5DC(U*)<br>C5DC(U)/C12<br>C5DC(U)/C14<br>C5DC(U)/C16<br>C5DC(U)/C18<br>C5DC(U)/C18:1<br>C5DC(U)/C2<br>C5DC(U)/Val<br>C5DC(U)/C0<br>C5DC(U)/C3<br>C5DC(U)/Cit<br>C5DC(U)/Met<br>C5DC(U)/Phe<br>C5DC(U)/Tyr<br>C5DC(U)/Xle<br>C5DC(U)/C10<br>C5DC(U)/C8<br>C5DC(U)/C5OH(U)<br>C5DC(U)/C18:2<br>C5DC(U)/C4<br>C5DC(U)/C5	Age<br>BW<br>Location	C3DC(U)/Orn<br>C3DC(U)/Ala	N:83<br><br>(Including 2 Dutch cases)
		**Low Markers**<br>C14:1/C5DC(U)<br>C10/C5DC(U)<br>C5/C5DC(U)<br>C6/C5DC(U)<br>C12:1/C5DC(U)			

*(U): (underivatized).

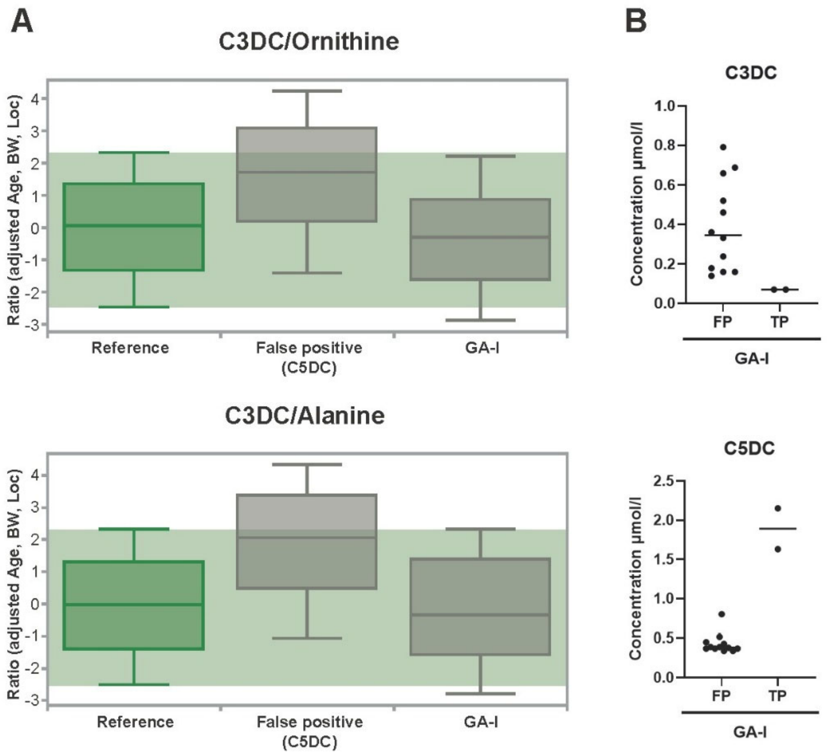

Figure S4. (**A**) Boxplot comparing the covariate corrected reference intervals (based on age, birthweight and location correction) of the C3DC/Ornithine ratio and the C3DC/Alanine ratio for the reference, false-positive C5DC, and GA-I-positive populations in CLIR. (**B**) Scatterplot indicating the con-centration of C3DC and C5DC for false-positive (FP) and true-positive (TP) cases of GA-I in the Dutch NBS program.

Table S4. configuration of the SCT for IVA.

Condition	Configuration	Included markers	Covariate corrections	Marker exceptions	Amount of cases included
IVA	Increasing<br>Weighted<br>Min/Max<br>Plot differentiators<br><br>Guidelines: Default Points	**High Markers**<br>C5<br>C5/C0<br>C5/C14<br>C5/C14:1<br>C5/C16<br>C5/C2<br>C5/C3<br>C5/C4<br>C5/C8<br>C5/Cit<br>C5/Met<br>C5/Phe<br>C5/Tyr<br>C5/Val<br>C5/Xle<br>4+5/2+3*	Age<br>BW<br>Location	N.A.	N:156
		**Low Markers**<br>C10/C5<br>C14:1/C5<br>C6/C5			

*4+5/2+3: (C4+C5)/(C2+C3).

Table S5. configuration of the SCT for FP C5.

Condition	Configuration	Included markers	Covariate corrections	Marker exceptions	Amount of cases included
FP C5	Increasing<br>Weighted<br>Min/Max<br>Bypass Tool runner<br>Plot differentiators<br><br>Guidelines: Default Points	**High Markers**<br>C5<br>C5/C14<br>C5/C18:1<br>C5/Cit<br>C5/Gly<br>C5/Met<br>C5/Phe<br>C5/Val<br>C5/Xle<br>C5/C16<br>C5/C18<br>C5/C8<br>C5/Pro<br>C5/Ala<br>C5/Arg<br>C5/C0<br>C5/C14:1<br>C5/C2<br>C5/C12<br>C5/C4	Age<br>BW<br>Location	C5/C2	N:51

		C5/C3<br>C5/Tyr<br>4+5/2+3*			
		**Low Markers**<br>C10/C5<br>C16:1OH/C5<br>(Xle/Ala)/C5<br>C14:1/C5<br>C6/C5<br>C16OH/C5			

*4+5/2+3: (C4+C5)/(C2+C3).

Table S6. configuration of the SCT for MSUD.

Condition	Configuration	Included markers	Covariate corrections	Marker exceptions	Amount of cases included
MSUD	Increasing Weighted Min/Max Plot differentiators<br><br>Guidelines: Default Points	**High Markers**<br>Xle<br>(X+V)/(P+T)*<br>Xle/Phe<br>Xle/Tyr<br>Xle/Ala<br>Val/Phe<br>Val	Age<br>BW<br>Location	-	N:96
		**Low Markers**<br>Met/Xle<br>C5/Xle<br>C4/Xle<br>C5/Val<br>C2/Xle<br>C3/Xle<br>C8/Xle<br>Tyr/Val<br>Met/Val<br>C0/Xle<br>Cit/Xle			

*(X+V)/(P+T): (Leucine + Valine) / (phenylalanine + tyrosine).
